# Evaluation of an automated method for arterial input function detection for first-pass myocardial perfusion cardiovascular magnetic resonance

**DOI:** 10.1186/s12968-016-0239-0

**Published:** 2016-04-08

**Authors:** Matthew Jacobs, Mitchel Benovoy, Lin-Ching Chang, Andrew E. Arai, Li-Yueh Hsu

**Affiliations:** National Heart, Lung, and Blood Institute, National Institutes of Health, Bethesda, MD USA; Department of Electrical Engineering and Computer Science, Catholic University of America, Washington DC, USA; Department of Biomedical Engineering, Ecole Polytechnique de Montreal, Montreal, Canada

**Keywords:** Cardiovascular magnetic resonance, Myocardial perfusion imaging, Arterial input function

## Abstract

**Background:**

Quantitative assessment of myocardial blood flow (MBF) with first-pass perfusion cardiovascular magnetic resonance (CMR) requires a measurement of the arterial input function (AIF). This study presents an automated method to improve the objectivity and reduce processing time for measuring the AIF from first-pass perfusion CMR images. This automated method is used to compare the impact of different AIF measurements on MBF quantification.

**Methods:**

Gadolinium-enhanced perfusion CMR was performed on a 1.5 T scanner using a saturation recovery dual-sequence technique. Rest and stress perfusion series from 270 clinical studies were analyzed. Automated image processing steps included motion correction, intensity correction, detection of the left ventricle (LV), independent component analysis, and LV pixel thresholding to calculate the AIF signal. The results were compared with manual reference measurements using several quality metrics based on the contrast enhancement and timing characteristics of the AIF. The median and 95 % confidence interval (CI) of the median were reported. Finally, MBF was calculated and compared in a subset of 21 clinical studies using the automated and manual AIF measurements.

**Results:**

Two clinical studies were excluded from the comparison due to a congenital heart defect present in one and a contrast administration issue in the other. The proposed method successfully processed 99.63 % of the remaining image series. Manual and automatic AIF time-signal intensity curves were strongly correlated with median correlation coefficient of 0.999 (95 % CI [0.999, 0.999]). The automated method effectively selected bright LV pixels, excluded papillary muscles, and required less processing time than the manual approach. There was no significant difference in MBF estimates between manually and automatically measured AIFs (*p* = NS). However, different sizes of regions of interest selection in the LV cavity could change the AIF measurement and affect MBF calculation (*p* = NS to *p* = 0.03).

**Conclusion:**

The proposed automatic method produced AIFs similar to the reference manual method but required less processing time and was more objective. The automated algorithm may improve AIF measurement from the first-pass perfusion CMR images and make quantitative myocardial perfusion analysis more robust and readily available.

## Background

First-pass contrast-enhanced perfusion cardiovascular magnetic resonance (CMR) is a useful diagnostic tool for the detection of coronary artery disease [1–3]. Quantitative assessment of myocardial blood flow (MBF) provides an accurate evaluation of myocardial ischemia, which appears promising for identifying coronary artery stenosis [4–8]. Quantitative assessment of MBF, however, requires the measurement of the arterial input function (AIF), which represents the transit of contrast through the left ventricular (LV) cavity [9]. Such AIFs are typically measured by manually drawing a region of interest (ROI) within the LV blood pool on a range of 45 to 90 perfusion images. These ROIs must be adjusted to account for motion from image to image to obtain the mean time-signal intensity curve. This manual process is time consuming, which may hinder quantitative assessment of large datasets. In addition, the manual analysis is subject to inter- and intra-operator variation. It has been shown that MBF estimates can be influenced by myocardial ROI contours tracing errors [10]. However, no detailed study has been conducted regarding how different AIF ROI selections influence MBF measurement.

Although automated AIF detection has been developed for cerebral perfusion MR, less effort has been made to automate AIF measurement from perfusion CMR. Carroll et al. [11] presented a method to measure the cerebral AIF by excluding late contrast arrival voxels and selecting the single voxel showing the largest signal intensity change. Peruzzo et al. [12] method discards voxels that poorly fit the expected cerebral AIF characteristics and classifies the remaining voxels with agglomerative hierarchical clustering to select the AIF voxels. Yin et al. [13, 14] presented two studies, one using hierarchical clustering and another using a normalized cut clustering scheme to select the final cerebral AIF cluster.

Several other automated AIF measurement methods have been presented in cerebral and tumor studies, but with a very limited sample size. Shi et al. presented an automated method applying to rat liver and human brain images [15]. Their method registers the images and applies a fast affinity propagation clustering algorithm for the AIF detection. Kim et al. also proposed an automatic method for use in mice skeletal tumors [16]. They used Kendall’s coefficient of concordance to identify regions of similar contrast dynamic curves for the AIF measurement. A combination of active contours and morphological image processing were also incorporated to improve the AIF detection.

Semi-automated AIF measurement has also been a popular research topic outside of the CMR field. Rijpkema et al. [17] proposed a two-step process of AIF detection for application to human tumors in the head and neck, prostate, and brain. Pixels with maximum contrast concentration greater than two standard deviations above the mean of the image are automatically thresholded and are interactively thresholded again using the arrival time of maximum concentration. Parker et al. [18] followed similar logic to extract AIF from brain, lung, and prostate images. They started by thresholding pixels whose maximum concentration arrival time were within 20 s of a manually determined contrast arrival time, and finished by selecting the pixels with peak concentration within the top 5 % of those remaining. This maximum concentration arrival time threshold was lowered to 10 s in a later work for use in humans with abdominal and pelvic tumors [19].

In addition, the extent to which different AIF ROI selections affect MBF measurement has not been thoroughly studied. Miller et al. [20] analyzed inter-operator variability by having two different people analyze all aspects of each perfusion CMR study, including myocardial and AIF ROI placement. They found a moderate agreement between MBFs from the two operators; however, this study did not quantify the effect of AIF variation from different operators since it was merely one of several uncontrolled variables in MBF quantification. Outside the CMR field, Cutajar et al. [21] tested the effect of two differently sized AIFs in renography. For each patient, two AIFs were measured from two standardized rectangular ROIs placed within the aorta, one was 12 voxels and the other was 30 voxels in area. These two AIFs were both used with the same kidney ROI to calculate renal perfusion and glomerular filtration rate, both of which were found to be significantly affected by the differently sized AIFs.

Another body of research has been focused on detecting contrast enhancement timing points in perfusion CMR applications. Zarninaba et al. [22] developed an automated method for detecting the start of contrast within the myocardium by sequentially deconvolving the AIF and tissue response curves under different contrast start times until a residual curve fit error cap was reached. The start of contrast resulting in the lowest residual curve fit error is selected. Breeuwer et al.’s method [23] detects a contrast enhancement time window for perfusion analysis. The start time of the window is selected as the point where the LV signal intensity is at a certain percentage of the LVs peak intensity, and the end time is placed at a fixed time offset after the myocardial peak intensity.

Relevant research has also been conducted on segmenting the myocardium from perfusion CMR images based on small datasets. Tarroni et al. [24] presented a semi-automated method to separate the heart into LV cavity and myocardial regions. Their method was a region-based level set technique that required a user-defined seed point in the LV cavity. Hautvast et al. [25] presented an automated method to detect the heart region and the myocardium. A bounding box similar to the one used in this study is created around the heart using Otsu thresholding on temporal maximal and minimal intensity projection images. The myocardium is segmented with a Hankel transform based ring detector and deformable template for refinement. It should be noted that due to the emphasis on myocardial segmentation in these works, their detected LV regions were based on myocardial image series and included papillary muscles in the cavity, and thus would not be optimal for AIF measurement.

In our previous work [26], an automated algorithm was presented to extract the LV blood cavity signal from perfusion CMR images using independent component analysis (ICA) [27] as a method to identify the LV and the right ventricle (RV).

This study presents an enhanced automated system for measuring the AIF from first-pass CMR images to assist quantitative analysis of myocardial perfusion. It is validated on a large clinical dataset using a dual-sequence technique for the AIF images [28]. We demonstrate that the automated approach is more robust, reproducible, and faster compared to the manual reference measurements. These results are calculated using several AIF quality metrics, such as signal intensity upslope, peak value, time to peak, full width at half maximum, and an *M*-value [13, 14]. Additionally, the automated algorithm is used to investigate the impact of different AIF ROI sizes on fully quantitative MBF estimates in the myocardial image series and are compared against those estimated using the manual reference AIF.

## Methods

### Image acquisition

A retrospective dataset consisting of 270 clinical perfusion CMR studies was analyzed. All studies were performed under procedures and protocols approved by the institutional review board of the National Heart, Lung and Blood Institute (NHLBI), and all subjects gave written informed consent (ClinicalTrials.gov Identifier: NCT00027170). Each study contained two perfusion imaging series, one at rest and one during vasodilator stress. Gadolinium-DTPA (Magnevist, Berlex Laboratories, Wayne, NJ, USA) was administered (0.05 mmol/kg) at 5 ml/s during stress and rest perfusion imaging followed by a saline flush.

**Fig. 1:**
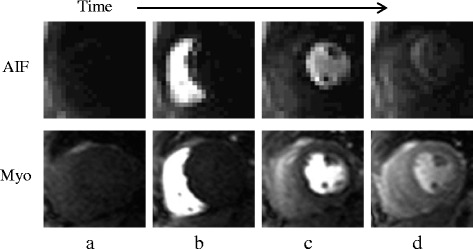
Dynamic contrast enhancement in perfusion CMR image series. The top row shows a low-resolution dedicated AIF series and the bottom shows a myocardial image series. **a** The baseline images show the heart without any contrast agent. As time passes, the contrast agent enters and enhances the **b** RV, **c** LV, and **d** myocardium

Perfusion CMR was performed on a 1.5 T scanner (Siemens Healthcare, Erlangen, Germany) with a saturation recovery dual-sequence technique [28] at every R-R interval over 60 heart beats. A steady-state free precession (SSFP) sequence was used in 245 studies; while a fast low-angle shot (FLASH) sequence was used in 25 studies. Typical imaging parameters for the myocardial perfusion image series included: 90° composite saturation preparation pulse, 50° (SSFP) or 12° (FLASH) flip angle, 90 ms (SSFP) or 100 ms (FLASH) inversion time, 1.2 ms echo time, 2.3 ms repetition time, 8 mm slice thickness, 360 × 270 mm field of view, 128 × 80 acquisition matrix, 256 × 192 image matrix after interpolation, and parallel imaging factor of 2 [29]. For each perfusion imaging, a FLASH low-resolution dedicated AIF image series was also acquired with a separate saturation pulse and typical parameters of 8° flip angle, 5.0 ms inversion time, 0.7 ms echo time, 1.3 ms repetitive time, 10 mm slice thickness, and 64 × 48 acquisition and image matrix size. Examples of both the low-resolution dedicated AIF image series and the myocardial series are shown in Fig. 1. At the start of each perfusion acquisition, two proton density (PD) weighted images were also acquired with no saturation preparation pulse for surface coil intensity normalization.

### Image processing algorithm

**Fig. 2:**
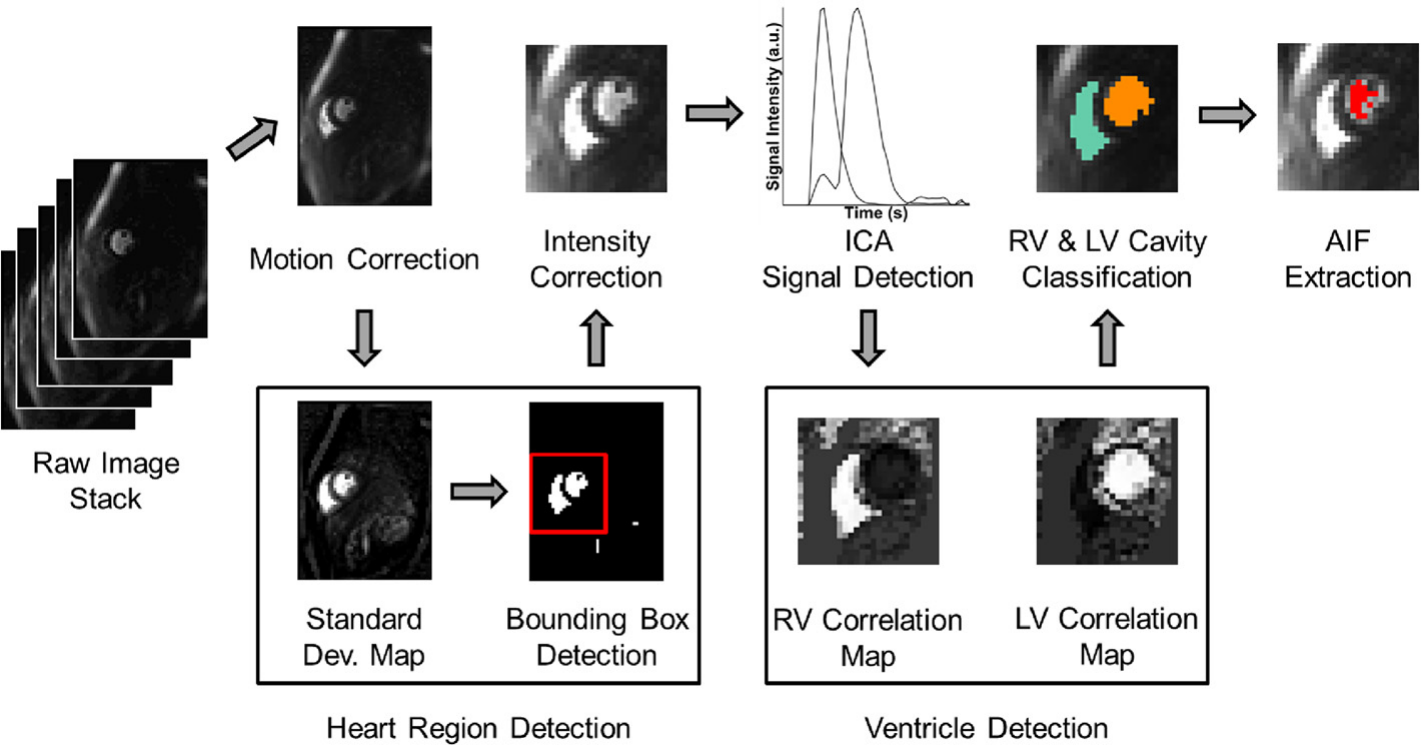
Automated image processing algorithm workflow. Raw images are first motion corrected, before the heart region is detected via segmentation of candidate ventricle regions from a standard deviation map. A bounding box is created around the selected regions, constraining further processing steps to this detected heart region. Intensity correction is performed prior to independent component analysis, which detects the time-intensity signal of the LV and RV. Correlation maps are used to classify each pixel to the RV, LV, or background (see Fig. 3). Finally, bright LV pixels are thresholded to extract the final AIF measurement (see Fig. 4)

Figure 2 shows the image processing pipeline of the proposed automated algorithm to measure AIFs from the image series. Note that the same algorithm is applicable to either dedicated AIF or standard myocardial image series.

#### Motion correction

Displacement of the heart can occur during image acquisition due to the patient’s cardiac or respiratory motion. A non-rigid body image registration technique based on optical flow computations is used to correct for anatomical structure motion invariably present in the perfusion image series [30]. This motion correction is applied to each series before subsequent image processing.

#### Heart region detection

The location of the heart region, in the form of a bounding box, is determined in order to aid further processing. This is achieved by identifying regions most indicative of the heart ventricles. Candidate ventricle regions are dynamically thresholded from a pixel-wise standard deviation map of the time series images. This standard deviation map highlights pixels with large intensity changes, such as those caused by contrast agent perfusion, while removing regions that remain constantly bright, such as the chest wall. This map is thresholded at one standard deviation above the mean for the AIF series, and at two standard deviations above the mean for the myocardial series to obtain the candidate ventricle regions. After binarization, regions that do not match the temporal signal characteristics of the ventricles are identified and removed. Specifically, regions whose intensity increase is less than twice their baseline intensity, indicating minimal contrast enhancement, are removed. Regions whose peak intensity occurs within the first or last 3 frames of the time series are also removed. Next, a similarity check is performed to examine whether each region represents a unique ventricular candidate. Similar regions are grouped as a single ventricular region that has been split by papillary muscles, image artifacts or slice placement. Similar regions are identified as having average time-signal intensity curves with a cross-correlation coefficient of more than 0.75, and whose minimum Euclidean distance is less than the sum of each region’s average radius. The final regions are subject to a linear voting scheme to iteratively determine which two candidate regions are most characteristic of the RV and LV cavities based on their time-signal features. Features used for voting classification include: distance to the image center, distance to previously selected candidate regions, size of each region, signal intensity upslope, peak value (PV), time to peak (TTP), full width at half maximum (FWHM), and an *M*-value [13, 14] which combines the previous three features as shown in Eq. 1.

1 $$ M=\frac{PV}{\left(TTP\ast FWHM\right)} $$

For each feature, the candidate ventricles are ranked by how well they match typical ventricle characteristics; that is those with larger region size, PV, upslope, *M*-value, smaller TTP, FWHM, and shorter distances to image center and to the previously selected ventricles. The ranks are converted to a score of 1 to *N*, 1 being the lowest rank and *N*, the number of candidate regions, the highest. The feature scores for each region are totaled, and the region with the highest total score is selected as a ventricle region. The second ventricle is selected similarly. The two selected ventricle regions are used to create a bounding box around the heart for subsequent processing (see Fig. 2).

#### Intensity correction

Surface coil intensity correction was based on the corresponding PD weighted images to improve the signal intensity uniformity of the heart region in the perfusion series. A slightly modified and automated version of the surface coil intensity correction algorithm presented in [31] is applied to reduce such inhomogeneity. Based on a hierarchical weighting scheme for foreground and background regions, this method estimates a polynomial signal intensity surface profile from the PD images which is used to adjust the signal intensity in the image series. After the surface coil intensity correction, the image series is further adjusted to remove baseline intensity based on pre-contrast perfusion images.

#### Ventricular pixel detection

**Fig. 3:**
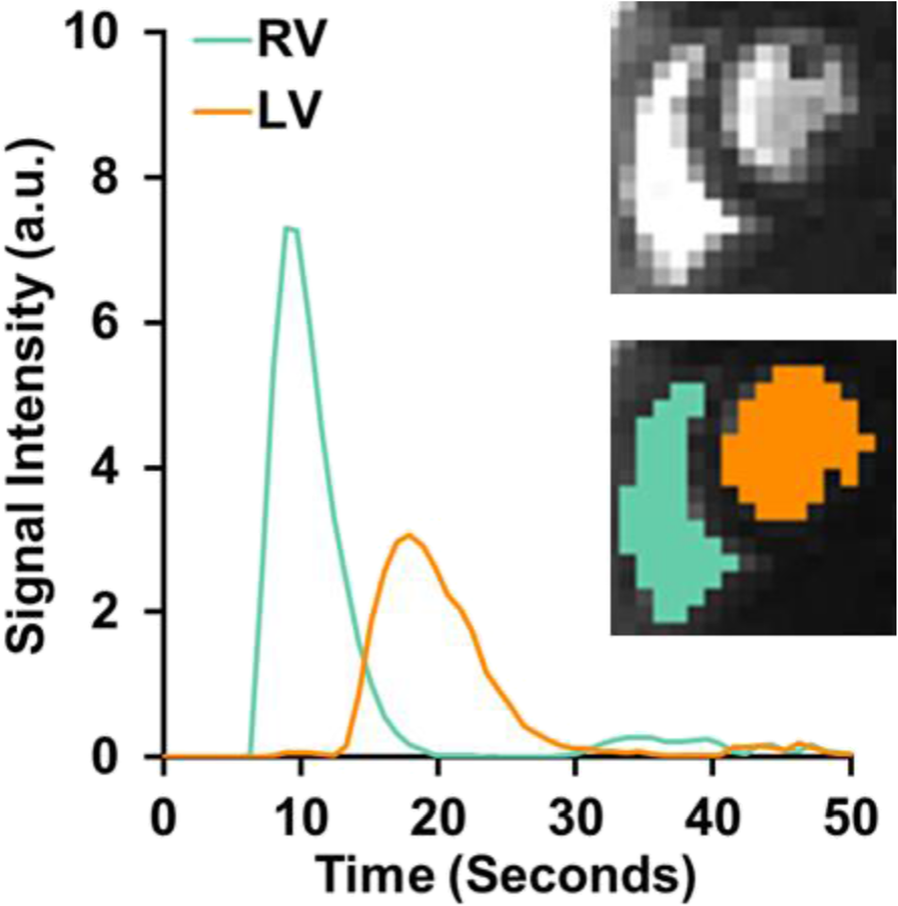
ICA detected RV and LV regions and their respective time-signal intensity curves. This intermediate step detects the maximum possible LV and RV pixels. Papillary muscles and potential partial volume errors will be excluded in a later step. Note that the detected RV signal is only used to differentiate the RV from the LV. The maximal intensity projection image of the series is displayed above the mask image for reference

To further the detection of ventricular blood pool pixels in the images, an ICA algorithm [27] is first used to obtain representative time-signal intensity curves from the previously identified ventricular regions. Assuming a mixture of two independent sources of signal (the RV and LV) in all ventricular regions, ICA separates and extracts the two primary time-signals that represent the dynamic contrast of the two ventricles. All of the pixels in the bounding box are classified to the RV, LV or background regions by computing their cross correlation to the RV and LV time-signals after the ICA process. Pixels with a cross correlation greater than a statistically determined value of 0.7 are assigned to the matching ventricle; the remaining pixels are then classified as the background region. The RV is identified as being the first region to reach peak intensity, which is followed by the LV region (see Figs. 2 and 3).

#### AIF extraction

**Fig. 4:**
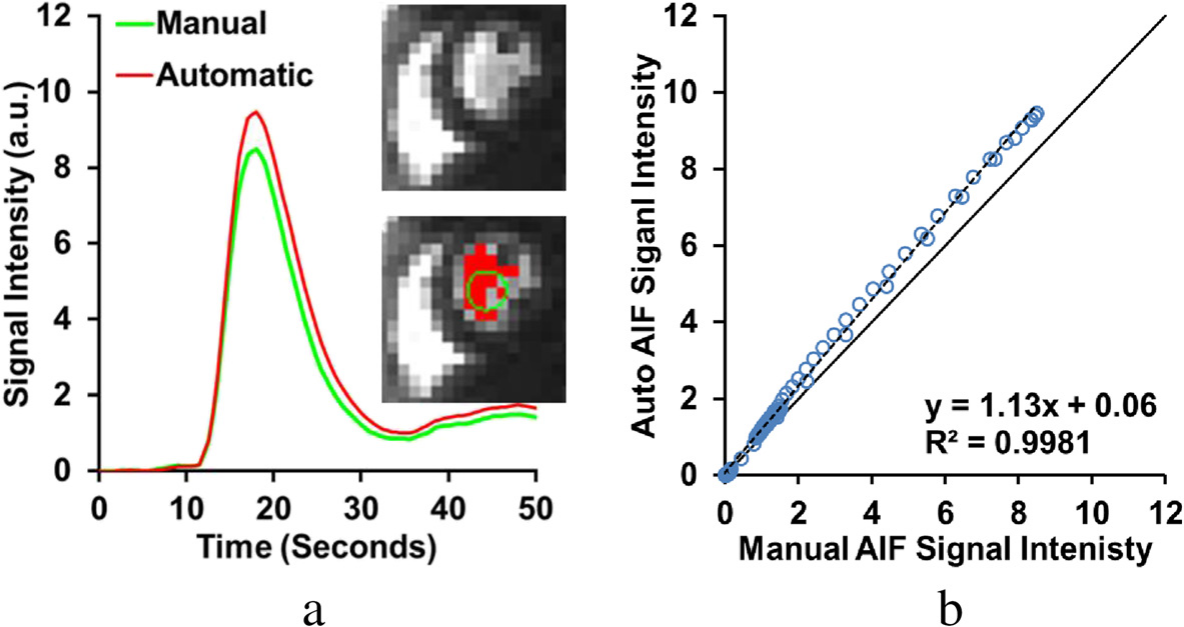
Comparison of AIF computed from automatically detected LV pixels vs. a manually selected ROI. The selected regions and the AIF curve measured from them are shown in **a**. Note how the automatically selected region (*red pixels*) excludes approximately three darker pixels included in the manual ROI (*green ring*), and includes other bright pixels outside the ring. The automatic AIF, as a result, displays a higher peak value and upslope, while maintaining similar time to peak and full-width at half maximum. Correlation of the manual and automatic AIF curves is shown in **b**. Despite the high correlation indicated, the differences in peak signal intensity give the trend line a larger slope (>1)

The final step of the algorithm is to select LV pixels that are brighter than a default threshold to compute an average intensity value of the blood pool in each image. This step excludes any papillary muscle pixels that may have been included in the LV region from the previous steps because they do not receive any contrast agent and remain dark. This step also excludes pixels that contain potential partial volume errors, as these pixels will also be darker than the average LV pixel. This closely replicates manual analysis, where the LV cavity is a relatively small but bright region within the heart. The default threshold was computed from the maximal intensity projection image as the 75^th^ percentile of the maximal intensity range of the LV region. Example results are shown in Figs. 2 and 4, where the red pixels indicate the pixels selected for the AIF measurement.

Finally, the AIF curve is linearly re-sampled at every half second to convert the time unit from image frames to seconds, since the perfusion imaging is performed on every R-R interval. We refer to this re-sampled curve as the AIF time-signal intensity curve for the remainder of the paper and use it in our statistical comparisons.

#### AIF timing point calculation

**Fig. 5:**
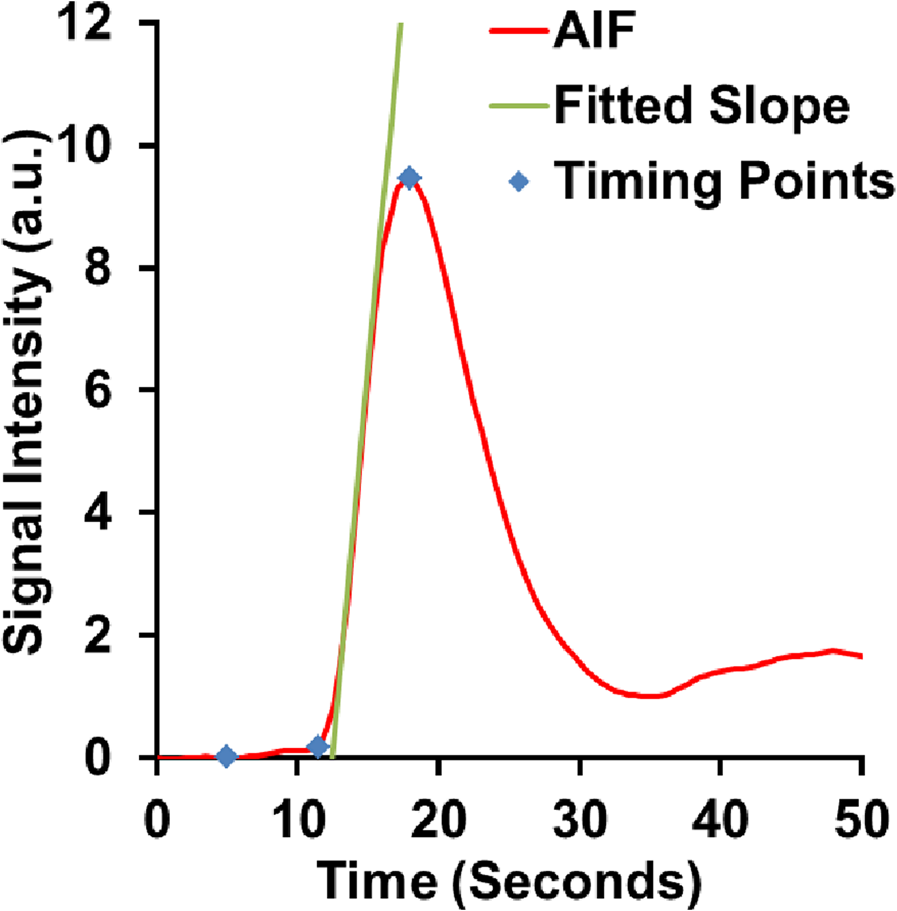
Automated AIF time point calculation example. The automatically detected timing points are displayed with blue markers; from left to right: baseline, start, and peak contrast enhancement. The green line shows the calculation of the start of contrast using a fitted slope line

In order to calculate important time-signal features for quantitative perfusion analysis as well as for candidate ventricle region selection, three contrast enhancement time points: baseline, start of, and peak contrast enhancement are derived from the AIF time-signal intensity curve automatically. First, the peak time is detected simply from the peak value of the time-signal intensity curve. The baseline time is determined next as the point of the curve with minimal intensity variation with its neighbors (the immediately adjacent points) between the beginning of the series and the rising peak (indicated by the point of maximal intensity change before the peak time). Finally the start time is detected by fitting a line to the rising peak and selecting the point of the curve geometrically closest to the intersection of this fitted line and the baseline intensity. As an example, automatically detected AIF timing points are shown in Fig. 5.

### Quantitative evaluation

Since many of the measurements made were not normally distributed, the median and 95 % confidence interval (CI) of the median were calculated throughout the analysis using SPSS Statistics software (International Business Machines Corp, Armonk, New York). A non-parametric Mann-Whitney U test [32] was used to determine if there were significant differences between the automated and manual AIF measurements, with *p* < 0.05 considered statistically significant.

#### AIF contrast enchantment characteristics comparisons

**Fig. 6:**
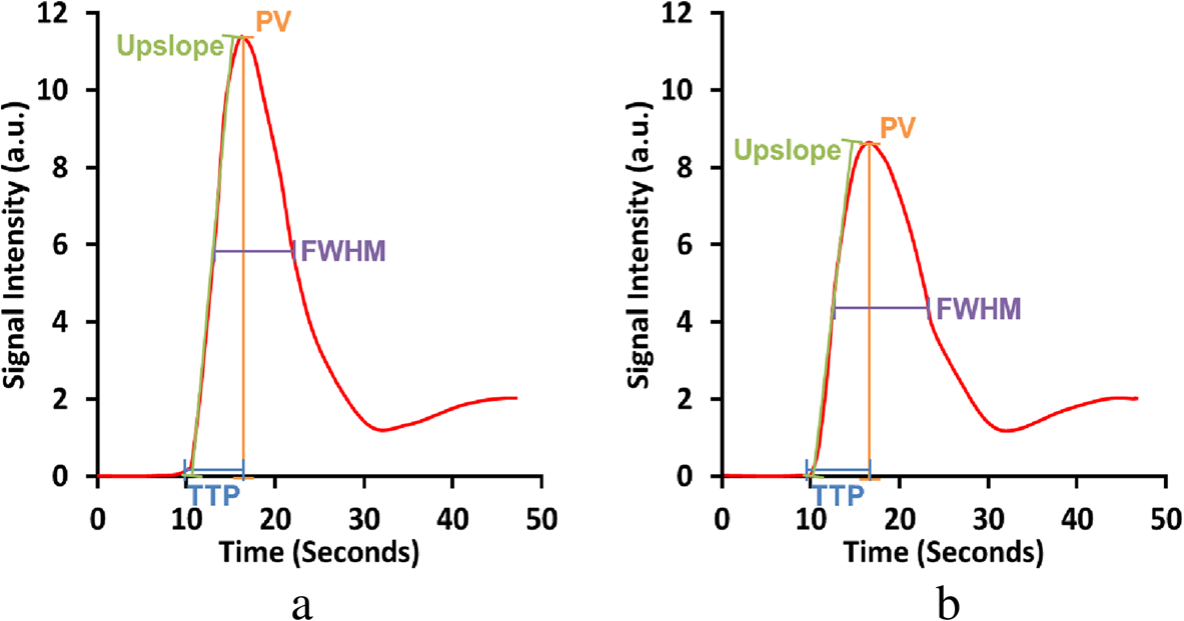
Quality metrics for evaluating different AIF curves measured from the same study. Curve **a** is more characteristic of a good AIF than curve **b** due to a higher peak value (PV), larger upslope, shorter time to peak (TTP), and thinner full-width at half-maximum (FWHM). The *m*-value (Eq. 1) is also higher for curve **a**

The performance of the proposed automated method was evaluated on both rest and stress image series from all clinical perfusion studies using custom image analysis software developed in Interactive Data Language (IDL, Exelis Visual Information Solutions, Boulder, Colorado). The results of the automated AIF detection were compared to a reference created by manually drawing an ROI in the LV blood cavity throughout the entire dataset. Pearson’s correlation coefficient and normalized root mean square error (RMSE) were used to evaluate the similarity between the automated and manual AIF time-signal intensity curves. Descriptive time-signal statistics of the AIF were compared which included TTP, FWHM, PV, signal intensity upslope, and *M*-value, as described in the previous sections. As shown in Fig. 6, accurate AIFs are characterized by high values of PV, upslope, *M*-value, and low values of TTP and FWHM [13, 14]. Such improvements will likely be due to selection of the brightest LV pixels, exclusion of papillary muscles, and exclusion of pixels containing partial volume errors.

#### AIF timing point and myocardial blood flow comparisons

Further evaluation was performed on a subset of 21 clinical studies consisting of dedicated AIF image series of normal healthy volunteers from the SSFP dataset. First, the automatically detected first-pass contrast timing point, the start of peak and peak time, from the automated AIF curve were compared against manually selected ones. Second, fully quantitative MBF estimates using the automatically and manually measured AIFs from the dedicated AIF image series were compared. To facilitate these comparisons, the endocardial and epicardial borders of the myocardium were manually traced by a trained CMR expert onto the myocardial image series using the custom software. The defined myocardial regions were subdivided into six equiangular sectors to derive myocardial time-signal intensity curves. The MBF calculation was based on a model constrained deconvolution technique [33]. To observe only the effect of the AIF upon the MBF, the manually selected timing points were used in all MBF calculations.

Next, MBF estimates were evaluated again with differently sized AIF ROI selections. Here we generated three differently sized ROIs by selecting three different percentile thresholds in the AIF extraction part of the automated algorithm (see “AIF extraction” section): the default ROI using the 75^th^ percentile, a larger ROI using the 50^th^ percentile, and the largest ROI using the 25^th^ percentile. MBF estimates were calculated using each of these AIFs as described previously.

Finally, the execution time required by the automated algorithm to extract the AIF was measured on these 21 studies using an Intel Core i7-3770 3.4GHz central processing unit (CPU). For comparison, the time required to manually perform the equivalent processing of the same input image series to measure the AIF using our custom software was also measured. The execution time for both automatic and manual processing included the time required for image loading, motion correction or registration, LV ROI selection, and AIF signal intensity calculation from the ROI. It does not include the myocardial contouring time. It should be noted that the custom image analysis software was specifically designed to facilitate quick and efficient manual ROI drawing and registration.

## Results

Two studies out of the initial 270 were excluded from the comparison: one due to a congenital heart defect and the other due to a contrast bolus administration issue. The automated method successfully measured the AIF from all of the remaining dedicated AIF series, and from all but four myocardial perfusion series resulting in a 99.63 % success rate. The automatically selected regions and measured AIF curves were verified visually for appropriateness.

Figure 2 shows sample intermediate results from the automated image processing algorithm. Figure 3 shows more detailed results of the automatic RV and LV cavity detection step, including the two detected ventricle regions and their respective time-signal intensity curves. It is important to note first that the detected RV region is merely used to exclude the RV pixels from AIF measurement. Second, in the detected LV region, papillary muscles have not yet been excluded at this stage, as it represents the maximum possible extent of the ventricular area which will be refined in the next intensity threshold step. Finally, Fig. 4 shows the AIF computed from the automatically selected LV pixels, after excluding papillary muscles and pixels with possible partial volume errors. As a comparison, the AIF measurement from the manually selected ROI in the LV is also shown.

**Fig. 7:**
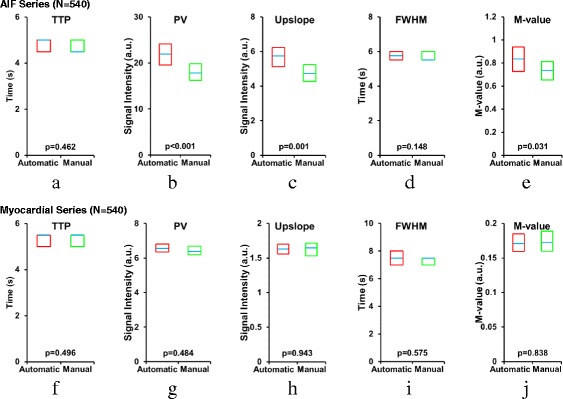
Comparison of automatically and manually measured AIF statistics using the quality metrics in Fig. 6. Dedicated AIF series statistics are presented in **a**–**e**, and myocardial perfusion series statistics are in **f–g**. Each box plot shows the median with a blue line and the 95 % confidence interval (CI) within a box for **a**, **f** TTP: time to peak, **b**, **g** PV: peak value, **c**, **h** upslope, **d**, **i** FWHM: full width at half maximum, **e**, **j** *M*-Value. The dedicated AIF series automatic PV, upslope, and *M*-value all are significantly higher than their manual counterpart, as shown by their *p*-values

The correlation coefficient between the manual and automated AIF time-signal intensity curves showed a median value of 0.999 (95 % CI [0.999, 0.999]). The RMSE between the manual and automated time-signal intensity curves had a median value of 3.33 %, 95 % CI [3.04 %, 3.73 %] over the whole dataset. To further evaluate the automated AIFs and compare with the manual references, Fig. 7 summarizes the statistical comparison of the AIF results using several quality metrics as shown in Fig. 6.

In the dedicated AIF image series, the temporal statistics of TTP and FWHM showed no significant difference between the two AIF measurements. However, the PV, signal intensity upslope, and *M*-value showed significantly higher values for the automated AIFs compared to the manual results (*p* < 0.001, *p* < 0.01, and *p* = 0.031 respectively). In the myocardial perfusion series, there was no significant difference between the manual and automatic statistics in any of the quality metrics.

**Fig. 8:**
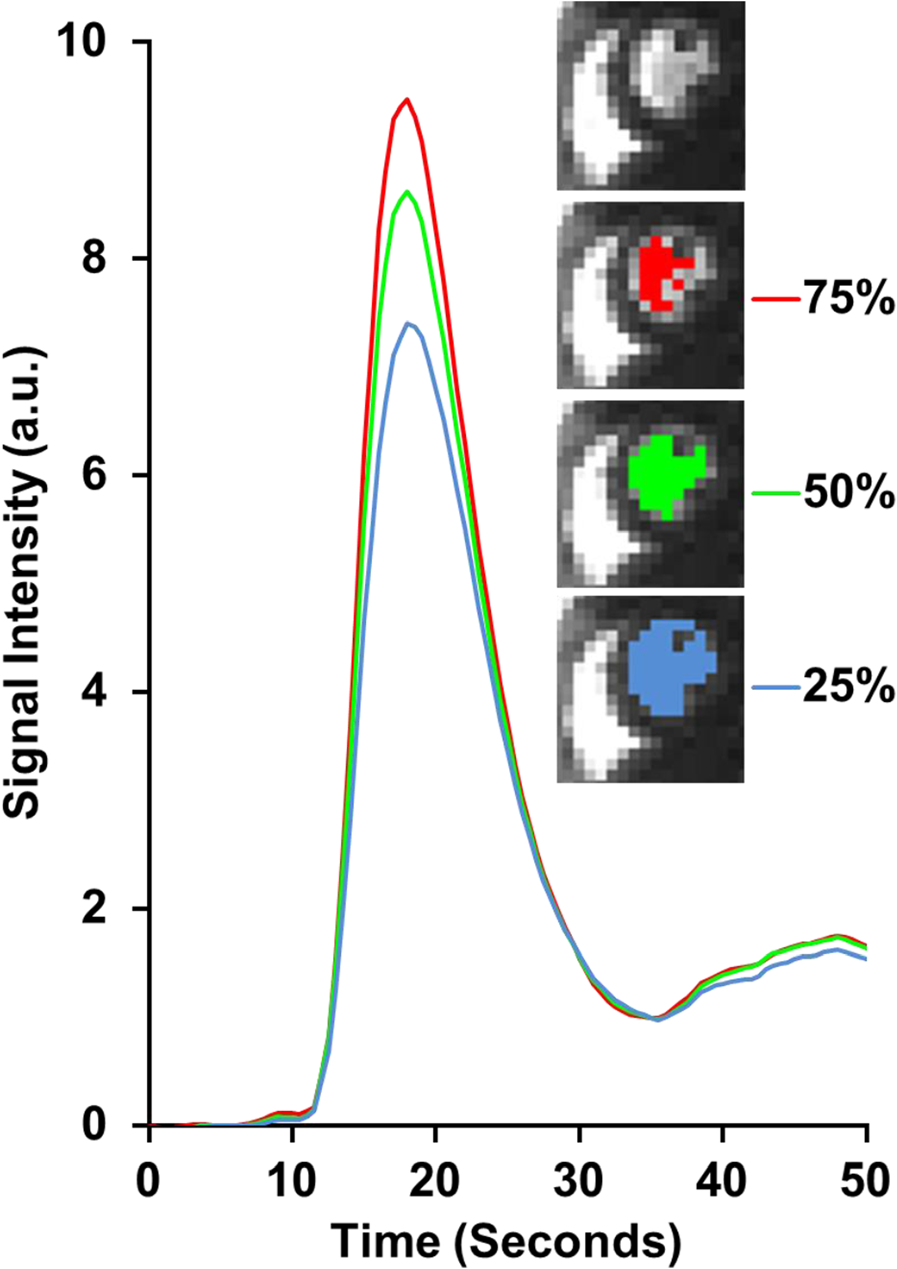
Comparison of the AIF measurements attained from three different intensity thresholds in the LV region. The regions correspond to three different intensity thresholds used during automatic AIF detection: The 75^th^, 50^th^ and 25^th^ percentile of the maximum LV intensity range. Note that the lower percentage of the threshold used, the more LV pixels were included and the lower the peak value of the AIF was measured

One important factor in the automated algorithm is the intensity threshold step in the LV region for the final AIF extraction. An example of different threshold settings (Fig. 8) demonstrates the trend that the use of a lower threshold will include more LV pixels and result in a lower AIF peak value.

On the evaluation of automatically detected AIF timing points, the differences between the automatically and manually selected timing points were not statistically significant. The median absolute difference of the start time of first-pass contrast was 0.5 s, 95 % CI [0.5, 0.5], while the median absolute difference of the peak time was 0.0 s, 95 % CI [0.0, 0.5] (both *p* = NS).

The MBF analysis results are presented in Table 1, including the manual reference standards and the proposed automatic method using the default 75^th^ percentile threshold. No significant difference was found between the MBF calculated using the manual reference AIF and the automatically measured AIF (both *p* = NS). Table 1 also shows a summary of the MBF measurements resulting from the three AIF threshold comparisons: the default 75^th^, and the alternate 50^th^ and 25^th^ percentile thresholds. There was no significant difference in rest series MBFs between the three threshold levels and the manual reference standard. Stress series, however, did show a significant increase of MBFs using the 25^th^ percentile threshold (*p* = 0.030), a directional change consistent with underestimating the peak of the AIF.

**Table 1:** Comparison of myocardial blood flow (MBF) estimates (in ml/g/min) using automated vs. manual AIF measurements

(ml/g/min)	Rest MBF	Stress MBF
Manual	1.11 [1.06, 1.16]	3.18 [3.01, 3.36]
Auto. 75 % (default)	1.14 [1.09, 1.20]	3.09 [2.96, 3.35]
Auto. 50 %	1.15 [1.09, 1.19]	3.21 [3.08, 3.41]
Auto. 25 %	1.17 [1.11, 1.23]	3.41 [3.22, 3.65]*

All results were calculated using AIFs measured from the dedicated AIF series. Results are expressed as the median and 95 % confidence interval. Automated results significantly different from the manual reference standard are marked with *(*p* < 0.05)

The median execution time of the automated method to process an image series was 26.1 s, 95 % CI [13.2, 27.1], while the manual methods required 102.2 s, 95 % CI [95.6, 114.8] (*p* < 0.001). It should be noted that the processing time of the algorithm is affected by image size; thus the dedicated AIF series required much less time (12.85 s, 95 % CI [12.61, 12.95]) than the myocardial perfusion series (27.5 s, 95 % CI [27.3, 27.6]). In either case, the automatic method is significantly faster than the manual (both *p* < 0.001).

## Discussion

We have presented an automated system for measuring the AIF from perfusion CMR image series. It has been shown to successfully process a wide array of perfusion series of varying conditions in a large clinical dataset. It has also been shown to be compatible with multiple imaging sequences. Automatically measured AIFs were found to be in agreement with manual measurements and AIFs measured from dedicated AIF image series produced similar MBF estimates. Furthermore, we have shown that while AIF and MBF measurements are robust to minor variation in blood cavity ROI selections, extreme differences can affect them, especially under stress. The automated system is capable of processing a large variety of image series faster than manual methods, provides consistently reproducible results, and effectively removes inter- and intra-operator variation in AIF measurement. These improvements may help make quantitative myocardial perfusion CMR analysis more objective and readily available to assist in the diagnosis of coronary artery disease.

Previous studies have suggested that accurate AIFs are normally characterized by high PV, large upslopes, and higher *M*-values [13, 14]. Our statistical comparisons (Fig. 7) indicate that significantly higher PV was measured in the dedicated AIF series by the automated system compared with manual ROIs. In addition, significantly higher signal intensity upslope, and *M*-values were measured by the automated system. The cause is the proposed method’s ability to detect the brightest LV pixels in the images, while also effectively excluding papillary muscle pixels and pixels with potential partial volume errors. Both of these are essential to avoid underestimation of the AIF peak signal and the upslope measurements. This also highlights the potential difficulties caused by inter- and intra-operator variation in manual measurements and the need for consistent and reproducible AIF measurements, which the automated method provides. Furthermore, in addition to being more reproducible, the execution time comparison also showed that the automated method is much faster than the manual method.

The differences between the automatically and manually selected AIF timing points were found to be negligible, indicating the automatic algorithm is in close agreement with manual analysis. It should be noted that the AIF time-intensity curves are sampled every 0.5 s, so the median error of the automatically detected start of first pass contrast was only 1 time point. In addition, the automatically detected peak point was virtually identical to the manually selected point.

The MBF analysis results (Table 1) show that the automatic method’s AIF produces similar results to manual measurements (*p* = NS for the default 75^th^ percentile threshold setting). The MBF measurements resulting from the three alternate ROI threshold comparisons (Table 1) indicate that the AIF is not sensitive to minor variations in ROI selection. The stress series MBF measurements was found to differ significantly from manual measurement, but only when selecting the largest tested AIF region (25^th^ percentile threshold). This extreme example, which may include potential partial volume errors or papillary muscles, emphasizes the necessity of excluding such pixels from the AIF measurement. It should also be noted that it is unlikely an experienced operator would draw such an ROI. Furthermore, despite the fact that our algorithm is applied to both the dedicated low resolution AIF and standard myocardial perfusion series, the MBF analysis was performed only using the AIF from the dedicated AIF series due to signal saturation in the blood pool of the myocardial series, which prohibits an accurate AIF measurement.

Two studies were excluded from our quantitative evaluation. The first was due to a suspected atrial septal defect which caused premature recirculation of the contrast agent and contaminated the first-pass. The second was classified as non-diagnostic due to inadequate contrast bolus administration. Both of these issues would prevent either a manually or automatically measured AIF from being usable for MBF quantification. Outside of these exclusions, the automated method successfully extracted the AIF from all AIF series and all but four myocardial series. The issues contributing to the detection failure included over segmentation in the standard deviation map and ICA signal separation error potentially resulting from imaging and residual motion artifacts.

Despite these few failed series, the automated method successfully extracted the AIF from the majority of the AIF and myocardial image series and overcame the main pitfall in manual AIF measurement: papillary muscle exclusion. Papillary muscles, which remain dark throughout the perfusion sequence, can cause underestimation of the AIF if included in the measurement region. In order to avoid these complex structures, an ROI may have to be made very small or irregular in shape which requires more time to draw manually. The automated method is at an advantage, as it can reliably detect the brightest pixels in the LV blood pool, which can be in either a connected or multiple discrete regions, to compute an AIF for MBF quantification.

### Limitations

The default 75 % LV intensity threshold in the final step of our algorithm was selected based on comparison with our reference standard manual AIF measurement. Another optimal threshold setting may be found if different datasets or multiple users were used to define the reference standard.

## Conclusion

We have presented an automated method to remove inter- and intra-operator variation and reduce processing time for measuring the AIF from first-pass perfusion CMR images. We have demonstrated that the automated approach produced similar AIF and MBF estimates to the reference manual method, but was more robust, reproducible, and faster. Our results also showed that different sizes of ROI selection in the LV cavity could change the AIF measurement and affect MBF calculation. The proposed method may improve AIF measurement from the perfusion CMR images and make quantitative myocardial perfusion analysis more robust and readily available.

### Ethics approval and consent to participate

All studies were performed under procedures and protocols approved by the institutional review board of the National Heart, Lung and Blood Institute (NHLBI), and all subjects gave written informed consent (ClinicalTrials.gov Identifier: NCT00027170).

### Consent for publication

All subjects gave consent for publication as part of their written informed consent (ClinicalTrials.gov Identifier: NCT00027170).

### Availability of data and materials

The dataset supporting the conclusions of this article can be made available in accordance with the Data Sharing Policy of the National Institutes of Health through a Material Transfer Agreement to protect the confidentiality of patient information.

## Abbreviations

AIF: arterial input function
AU: arbitrary units
CI: confidence interval
CMR: cardiovascular magnetic resonance
CPU: central processing unit
FLASH: fast low-angle shot
FWHM: full width at half maximum
ICA: independent component analysis
IDL: interactive data language
LV: left ventricle
MBF: myocardial blood flow
NHLBI: National Heart, Lung and Blood Institute
NS: not statistically significant
PD: proton density
PV: peak value
RMSE: root mean square error
ROI: region of interest
RV: right ventricle
SSFP: steady-state free precession
TTP: time to peak
